# Beyond the Cornea: Systemic Diseases and Their Impact on Endothelial Health—A Narrative Review

**DOI:** 10.3390/jcm15083013

**Published:** 2026-04-15

**Authors:** Maria-Emilia Cerghedean-Florea, Cosmin Adrian Teodoru, Horațiu Dura, Mihai Dan Roman, Adrian Hașegan, Adrian Boicean, Mihaela Laura Vică, Horia Stanca, Ciprian Tănăsescu

**Affiliations:** 1 Faculty of Medicine, “Lucian Blaga” University of Sibiu, 550024 Sibiu, Romania; mariaemilia.florea@ulbsibiu.ro (M.-E.C.-F.);; 2 Department of Cellular and Molecular Biology, “Iuliu Haţieganu” University of Medicine and Pharmacy, 400012 Cluj-Napoca, Romania; 3 Institute of Legal Medicine, 400006 Cluj-Napoca, Romania; 4 Department of Ophthalmology, “Carol Davila” University of Medicine and Pharmacy, 050474 Bucharest, Romania

**Keywords:** corneal endothelium, endothelial dysfunction, diabetes mellitus, chronic kidney disease, autoimmune disease

## Abstract

**Background/Objectives**: The corneal endothelium maintains corneal transparency through its barrier function and active pumping mechanism that regulates stromal hydration. Limited regenerative capacity makes these cells vulnerable to progressive cell loss. Although local ocular factors are well known, recent data suggest that numerous systemic diseases may contribute to endothelial dysfunction and reduce endothelial reserve before the onset of clinically apparent corneal pathology. The purpose of this narrative review is to synthesize current evidence on the impact of systemic diseases on corneal endothelial health and to highlight the underlying mechanisms and clinical implications. **Methods**: A narrative literature review was conducted using the PubMed, MEDLINE, and Google Scholar databases for articles published between January 2000 and December 2025. Observational studies, case series, and review articles that evaluated structural or functional changes in the corneal endothelium in association with systemic diseases were included. **Results**: Reviewed literature shows that several categories of systemic diseases are associated with signs of corneal endothelial stress. These changes include decreased endothelial cell density, increased cell size variability, reduced hexagonality, and, in some cases, increased central corneal thickness. Metabolic, cardiovascular, renal, autoimmune, and hypoxic conditions, as well as extracellular matrix disorders and aging, show consistent associations with these changes. **Conclusions**: Systemic diseases can compromise corneal endothelial integrity and reduce functional reserve even in the absence of clinically evident corneal pathology. Recognition of these associations underscores the importance of evaluating the patient’s systemic context, including a detailed medical history and corneal endothelial analysis, particularly before intraocular surgery.

## 1. Introduction

**Figure 1:**
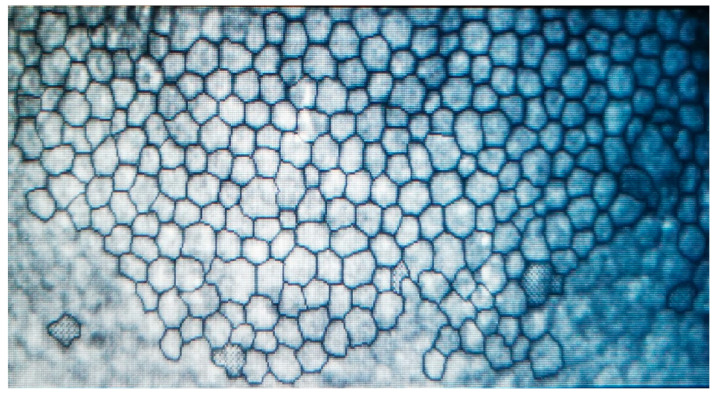
Endothelial corneal cells on specular microscopy.

The cornea is a transparent, avascular tissue, an important refractive medium that allows light to pass through to the retina. Its transparency is due to the precise arrangement of collagen fibers and controlled hydration of the tissue [1]. It consists of several layers, including the epithelium, Bowman’s layer, the pre-Descemet layer (Dua), Descemet’s membrane, and the endothelium. The corneal endothelial cell (CEC) layer is approximately 5 µm thick and consists of a single layer of polygonal, predominantly hexagonal cells that play an essential role in maintaining corneal transparency and normal visual function (Figure 1). Balance is ensured by a dual mechanism, the semipermeable barrier function and the activity of an energy-dependent pump that actively removes excess fluid from the corneal stroma [2,3].

Throughout life, CEC density progressively decreases [4]. Maximum density is at birth (approximately 3000 cells/mm^2^), then decreases by approximately 0.6% per year, reaching approximately 2500 cells/mm^2^ in adulthood [5,6,7]. These cells do not proliferate or regenerate; instead, they stay stopped in the G1 phase of the cell cycle [8]. Following cell loss, the corneal endothelium initially compensates through migration and enlargement of the remaining cells, accompanied by increased morphological heterogeneity. However, when endothelial cell loss exceeds the compensatory capacity and the remaining cell population becomes insufficient, corneal endothelial decompensation may occur, leading to visual impairment that may become irreversible.

Beyond its structural organization, the cornea functions as an integrated neurobiological unit. It is one of the most densely innervated tissues in the body, and corneal nerves are involved not only in pain perception but also in maintaining the homeostasis of the ocular surface. They regulate epithelial integrity, tear secretion, the blink reflex, healing processes, and neuro-immune balance [9,10,11]. Furthermore, this neuro-corneal axis is supported by a complex trophic microenvironment, in which nerve fibers interact with Schwann cells and limbal stem cells, contributing to epithelial renewal and the cornea’s ability to regenerate after injury [10,11]. In this context, systemic diseases can affect the cornea not only through metabolic or vascular mechanisms, but also by disrupting neurotrophic support, reducing the cornea’s ability to respond to stress. This perspective is also relevant to the vulnerability of the corneal endothelium, as endothelial functional reserve can be influenced by global imbalances in corneal homeostasis [9,10,11]. From a clinical perspective, understanding the mechanisms that lead to corneal endothelial cell loss remains a challenge, particularly in the context of systemic influences that are insufficiently characterized. Corneal decompensation occurs when cell density falls below a critical threshold, estimated at approximately 500 cells/mm^2^, at which point corneal transparency can no longer be maintained [12]. Although local ocular factors have been well studied, the contribution of systemic diseases to endothelial dysfunction remains incompletely understood. Systemic metabolic and vascular disorders may induce subclinical endothelial stress, potentially reducing endothelial reserve before overt corneal pathology becomes clinically apparent. Therefore, this narrative review critically examines the existing evidence on the impact of systemic diseases on corneal endothelial health. It discusses shared pathophysiological mechanisms, gaps in the current literature, and relevant clinical implications for patient evaluation and management, with the aim of promoting an integrated perspective that extends beyond a purely corneal approach.

## 2. Materials and Methods

A narrative review of the literature was conducted to analyze the impact of systemic diseases on the corneal endothelial cell layer. A structured search was conducted using PubMed, MEDLINE, and Google Scholar, including articles published between January 2000 and December 2025, with the aim of encompassing both foundational and recent evidence.

The search strategy used combinations of general and specific terms such as “corneal endothelium”, “endothelial cell density”, “endothelial dysfunction”, and “corneal decompensation”, along with terms related to major categories of systemic diseases and underlying mechanisms, such as “diabetes mellitus”, “cardiovascular disease”, “chronic kidney disease”, “autoimmune disease”, “oxidative stress”, “chronic inflammation”, and “metabolic dysfunction”.

All relevant clinical studies, including observational studies, case series and review articles, were considered. The search was limited to publications in English. Given the narrative nature of this review, studies were selected based on their clinical relevance and contribution to understanding pathophysiological mechanisms, without applying strict inclusion or exclusion criteria, but with emphasis on methodological quality and consistency of findings. Data extraction focused on the type of systemic disease, proposed mechanisms of endothelial injury, reported changes in endothelial cell density and morphology, and associated clinical implications.

Artificial intelligence tools were used exclusively for language editing and paraphrasing of the manuscript. These tools did not contribute to literature selection, data analysis, or interpretation of results (ChatGPT, OpenAI, San Francisco, CA, USA; GPT-5.2).

## 3. Systemic Diseases and Corneal Endothelial Dysfunction

### 3.1. Metabolic Disorders

Metabolic disorders represent a heterogeneous group of conditions in which chronic hyperglycemia plays a central, though not exclusive, role in tissue damage. Beyond the disruption of carbohydrate metabolism, alterations in lipid metabolism also appear to contribute to the vulnerability of the corneal endothelium. Experimental data have shown that hyperlipidemia can impair the integrity of intercellular junctions, reduce pump function, and induce oxidative stress in the corneal endothelium [13]. More recently, it has been suggested that, in diabetes, altered cholesterol biosynthesis in corneal endothelial cells, via the SREBF2/SQLE pathway, may further contribute to endothelial dysfunction and increased susceptibility to corneal edema [14]. Chronic hyperglycemia is a hallmark of diabetes mellitus, a metabolic condition brought on by either insufficient insulin secretion, impaired insulin action, or both. The most common ocular complication of diabetes is diabetic retinopathy, but the disease’s effects extend beyond the retina [15]. Studies have reported morphological changes in corneal endothelial cells in patients with diabetes, including reduced endothelial cell density and hexagonality, increased pleomorphism, polymorphism, and central corneal thickness (CCT) [16,17]. Structural or functional alteration of the corneal endothelium reduces the functional reserve of the cornea and may increase the risk of decompensation. Under these conditions, the cornea becomes more susceptible to complications, especially after trauma or surgical procedures [18,19].

The function of corneal endothelial cells is mainly based on the Na^+^/K^+^-ATPase pump, which maintains corneal transparency by regulating fluid transport from the stroma to the aqueous humor. In patients with diabetes mellitus, chronic hyperglycemia reduces Na^+^/K^+^-ATPase activity and intensifies aldose reductase activity, with intracellular sorbitol accumulation [20,21]. Within the polyol pathway, excess glucose is converted to sorbitol by aldose reductase, and the intracellular accumulation of sorbitol, acting as an osmolyte, induces osmotic stress and cellular swelling. In addition, increased flux through this pathway leads to depletion of NADPH, impairing antioxidant defenses and promoting the generation of reactive oxygen species (ROS) [22]. Elevated glucose levels cause increased mitochondrial production of reactive oxygen species, leading to nuclear DNA damage and activation of poly(ADP-ribose) polymerase. In this context, oxidative stress further impairs mitochondrial function and contributes to cellular damage in the corneal endothelium, altering its physiology [23,24].

In addition to endothelial alterations, diabetes mellitus is also associated with a spectrum of corneal abnormalities collectively referred to as diabetic keratopathy. These include impaired epithelial wound healing, increased corneal thickness, reduced corneal sensitivity and endothelial dysfunction [21]. The existing data largely support the existence of an association between diabetes mellitus and changes in the corneal endothelium, although the results are not entirely consistent. In this regard, case–control studies have shown a decrease in endothelial cell density in these patients compared to healthy subjects, often accompanied by increased cell size variability and reduced hexagonality [25].

Regarding the type of diabetes, more pronounced endothelial changes have been described in type 1 diabetes. Módis et al. reported a significant decrease in endothelial cell density and an increase in central corneal thickness, with these changes being correlated with HbA1c values and disease duration, while in patients with type 2 diabetes no significant endothelial differences were observed [26]. Also, in children and adolescents with type 1 diabetes, the reduction in endothelial cell density and increase in corneal thickness were correlated with disease duration [27]. On the other hand, patients with long-standing type 2 diabetes also showed lower endothelial cell density and increased corneal thickness compared to newly diagnosed patients and subjects without diabetes [28]. A decrease in endothelial cell density in type 1 diabetes has been described independently of the presence of diabetic retinopathy [29].

Changes in endothelial morphology have also been documented. In children and adolescents with type 1 diabetes, increased cell size variability and reduced hexagonality have been observed [30]. Similar changes have also been reported in diabetic patients analyzed according to disease duration, suggesting that endothelial damage may occur regardless of diabetes chronicity [31]. In type 2 diabetes, some studies have reported reduced endothelial cell density and increased corneal thickness, with no clear correlations with HbA1c or disease duration [32]. Other investigations have confirmed reduced density and increased variability in cell size in diabetic patients compared to the control group [33], and longer duration of diabetes has been associated with more pronounced morphological changes, including increased polymegathism [34]. Overall, most studies indicate that diabetes is associated with reduced corneal endothelial cell density and variable morphological changes, although the magnitude of these alterations differs according to diabetes type, disease duration, and glycemic control [25,26,27,28,29,30,31,32,33,34]. More recent prospective data further support these observations. A study evaluating patients with well-controlled type 2 diabetes reported significantly lower endothelial cell density compared to non-diabetic controls, despite adequate metabolic control and the absence of advanced diabetic retinopathy [35]. These findings suggest that corneal endothelial impairment may occur early in the course of diabetes and may not be fully prevented by optimal glycemic control.

Overall, these changes can be explained by several interconnected mechanisms. Chronic hyperglycemia leads to oxidative stress and metabolic imbalances that directly affect corneal endothelial cells. At the same time, mitochondrial dysfunction and decreased ATP production reduce the efficiency of the endothelial pump. Furthermore, the accumulation of toxic products and redox imbalance promote cellular damage. Together, these processes lead to decreased cell density, morphological changes, and a reduction in the functional reserve of the endothelium.

### 3.2. Cardiovascular Disease

Cardiovascular and cerebrovascular illnesses have been progressively explored in relation to corneal endothelial function. Although the cornea is avascular, emerging data suggest that its endothelium may mirror systemic vascular pathology. In patients with transient ischemic attack and minor ischemic stroke, corneal confocal microscopy demonstrated reduced endothelial cell density together with increased cell size compared with healthy controls [36]. Comparable findings were described in patients evaluated after acute ischemic stroke, who exhibited a significant decline in endothelial cell density together with increased endothelial cell size [37]. At a population level, a large global survey and meta-analysis including 39,762 eyes from 42 countries identified an inverse association between endothelial cell density and mortality from cardiovascular diseases, particularly coronary artery disease and hypertension [38].

A case–control study evaluating patients with primary arterial hypertension, stratified according to disease severity, demonstrated morphological alterations of the corneal endothelium compared with healthy subjects. Although endothelial cell density and mean cell area did not differ significantly between groups, the coefficient of variation in cell size was significantly increased and the percentage of hexagonal cells was reduced in hypertensive patients. Moreover, these changes were dependent on hypertension grade, with progressive increases in polymegathism and decreases in hexagonality as disease severity increased [39]. A retrospective analysis of patients who developed corneal endothelial insufficiency after cataract surgery showed that hypertension and cerebrovascular disease were significantly more common in those with postoperative endothelial failure compared to patients without decompensation. This suggests that chronic vascular pathology may reduce endothelial reserves and increase susceptibility to surgical stress [40].

In addition to hypertension and stroke, there are other cardiovascular risk factors that appear to influence the integrity of the corneal endothelium. In this regard, patients with endothelial dystrophy have been found to have an increased prevalence of cardiovascular disease, supporting the concept that the corneal and vascular endothelium share common biological characteristics [41,42].

Smoking and hyperlipidemia have also been associated with endothelial alterations. Experimental and clinical data indicate that hyperlipidemia disrupts intercellular junctions, impairs pump function, reduces endothelial cell density, and decreases hexagonality in a severity-dependent manner [43,44,45]. Furthermore, a recent systematic review with meta-analysis reported lower endothelial cell density in smokers compared with non-smokers, suggesting that chronic hypoxia and systemic inflammation may adversely affect corneal endothelial structure [46].

These changes may be related to systemic vascular impairment and the chronic inflammation associated with cardiovascular disease. In this context, reduced blood flow and oxidative stress can influence cellular metabolism and lead to alterations in the structure and function of the corneal endothelium.

### 3.3. Chronic Kidney Disease (CKD)

Chronic kidney disease is a progressive condition characterized by a decline in kidney function with the onset of systemic metabolic and inflammatory disorders [47,48]. Major pathophysiological mechanisms involved in ocular damage include vascular remodeling, persistent inflammation, increased oxidative stress, endothelial dysfunction, and accelerated progression of atherosclerosis [47]. These systemic changes can directly affect the stability of the corneal endothelium, which is a tissue dependent on metabolic homeostasis and particularly vulnerable to oxidative and inflammatory stress.

Multiple studies have evaluated corneal endothelial changes in patients with chronic kidney disease by comparing dialysis patients, non-dialysis patients, and healthy control groups. In most studies, morphological changes were observed, even though endothelial cell density remained relatively preserved [49,50,51]. Kanawa et al. reported similar cell density values between groups, but with a significant increase in the coefficient of variation and a reduction in hexagonality, suggesting polymegathism and pleomorphism in patients with CKD [49]. Comparable results were also described by Dube et al., who highlighted changes in cell variability and average cell size, with no consistent differences in cell density between groups [50]. Also, similar data were reported by Gupta et al., who confirmed an increase in the coefficient of variation and altered endothelial morphology in patients with CKD, regardless of disease stage [51].

On the other hand, Sati et al. demonstrated a significant reduction in cell density and an increase in central corneal thickness in dialysis patients compared to non-dialysis patients and the control group, suggesting a more pronounced impact of advanced disease and uremic status on the corneal endothelium [47]. Mahmoud et al. also reported a significant decrease in cell density and hexagonality in the dialysis patient group, associated with an increase in cell variability. The study results also showed a negative correlation between cell density and urea and creatinine values [52].

With regard to the relationship with biological parameters, the results are heterogeneous. Some studies have not identified correlations between urea or creatinine and endothelial parameters [49,50,51], while others have highlighted a negative association between the levels of these markers and cell density [47,52]. Overall, available data suggest that CKD is consistently associated with morphological changes in the corneal endothelium, while reduced cell density occurs more frequently in dialysis patients or in advanced stages of the disease [47,49,50,51,52]. These findings suggest that alterations in the corneal endothelium in chronic kidney disease are not solely due to structural damage, but also to systemic metabolic imbalances and uremic toxicity. It is possible that chronic exposure to inflammatory mediators and oxidative stress contributes to subclinical endothelial dysfunction, which may precede detectable cell loss and becomes clinically relevant under conditions of additional stress, such as intraocular surgical procedures.

### 3.4. Immune-Mediated and Autoimmune Disorders

**Graves’ orbitopathy (GO)**

GO is an organ-specific autoimmune disorder associated with Graves’ disease, characterized by orbital inflammation and tissue remodeling. Activation of orbital fibroblasts by autoantibodies leads to cytokine release, extracellular matrix expansion, and adipogenesis, processes that result in proptosis and increased exposure of the ocular surface. In this context, the inflammatory environment and instability of the ocular surface can generate stress on corneal structures, including the endothelium [53].

A cross-sectional study involving 101 eyes from 55 patients with GO evaluated corneal changes using in vivo confocal microscopy. Patients with GO showed signs of corneal inflammation and structural changes in the ocular surface. An increased number of activated keratocytes and changes in the corneal nerve plexus were observed. These changes were more pronounced in active forms of the disease [54].

Similarly, Zhou et al. analyzed the corneal endothelium using specular microscopy and reported changes in endothelial cell morphology in patients with GO. The most relevant change was an increase in the coefficient of variation (CV) of the cell surface area, a marker of endothelial polymegathism and cellular stress. This change was more evident in active forms of the disease and correlated positively with the clinical activity score, suggesting an association between active inflammation and morphological instability of the endothelium [53]. Similar results were reported by Oklar et al., who highlighted endothelial morphological changes characterized by an increase in the coefficient of variation of cell area and standard deviation of cell area, as well as a reduction in the percentage of hexagonal cells. These changes were more pronounced in active GO and correlated with the clinical activity score of the disease [55]. Together, these data suggest that corneal endothelial damage in GO is predominantly manifested by cellular morphological instability, which may reflect the impact of disease-associated inflammation on endothelial health.

**Sjögren’s syndrome**

Sjögren’s syndrome is a systemic autoimmune disease associated with chronic ocular inflammation and severe dry eye. Available data suggest that corneal involvement in this condition is not limited to the epithelium and tear film but may also affect the corneal endothelium. Proposed mechanisms include loss of neurotrophic support, activation of the local immune response, and cytotoxic stress induced by inflammatory mediators, which may contribute to a decrease in corneal endothelial cell density [56,57]. Chronic ocular inflammation can cause endothelial cell loss through several mechanisms. These include leukocyte activation and adhesion to the corneal endothelium, release of inflammatory mediators (cytokines, chemokines, and metalloproteinases), and induction of oxidative stress and endoplasmic reticulum stress. Furthermore, damage to the corneal nerves can lead to reduced neurotrophic support, further contributing to endothelial damage [58]. Also, El-Kady et al. have shown that patients with moderate or severe dry eye present a significant reduction in corneal endothelial cell density compared with healthy subjects, a change that correlates with disease severity [59]. However, evidence directly investigating corneal endothelial cell alterations in Sjögren’s syndrome remains limited.

**Rheumatoid arthritis**

The most common ocular manifestations in rheumatoid arthritis are keratoconjunctivitis sicca, episcleritis, scleritis, and peripheral ulcerative keratitis. However, systemic inflammation and inflammatory mediators can alter the ocular microenvironment with an impact on the corneal endothelium [59]. A study by Tasli et al. showed that patients with rheumatoid arthritis show signs of endothelial stress, characterized by increased cell size variability and a reduced percentage of hexagonal cells. Although endothelial cell density did not differ significantly from that of healthy subjects, these morphological changes suggest a compensatory cellular response. In addition, the authors observed a negative correlation between disease activity and endothelial cell density, indicating that more intense inflammation may be associated with a greater degree of endothelial damage [60]. Other studies suggest that corneal damage in rheumatoid arthritis is not limited to the endothelium. Using in vivo confocal microscopy, Villani et al. showed microstructural changes in the cornea. These included thinning of the stroma and an increase in the number of activated keratocytes, as well as alterations in the subbasal nerve plexus. Furthermore, the number of activated keratocytes and beadlike formations correlated with systemic disease activity, suggesting that these corneal changes may reflect the intensity of the systemic inflammatory process [61].

**Systemic lupus erythematosus**

Although the most common ocular manifestation in systemic lupus erythematosus is keratoconjunctivitis sicca, inflammation can also affect the deeper layers of the cornea, including the endothelium [62,63]. El-Kady et al. reported that patients with systemic lupus erythematosus show a significant decrease in endothelial cell density and a reduction in central corneal thickness compared to healthy subjects, with no significant changes in cell size variation or hexagonal cell percentage, suggesting possible endothelial impairment associated with systemic inflammation [59]. In addition to the inflammatory mechanisms associated with the disease, the treatment used for lupus may contribute to corneal changes. In a study evaluating lupus patients undergoing long-term treatment with hydroxychloroquine, a decrease in endothelial cell density and changes in corneal parameters were observed compared to healthy controls, suggesting that both the disease and therapy may influence the corneal endothelium [62,64].

**Dry eye disease**

Dry eye syndrome is one of the most common conditions affecting the ocular surface and is now recognized not simply as a disorder of the tear film, but as a disease characterized by hyperosmolarity, chronic inflammation, and a loss of immune homeostasis in the ocular surface [65,66]. Although the primary involvement affects the corneal epithelium and the tear film, recent data suggest that changes caused by dry eye may extend beyond the superficial layers of the cornea and may also involve the corneal endothelium [67,68].

A clinical study conducted by Kheirkhah et al. demonstrated that patients with moderate to severe dry eye have a significantly lower density of corneal endothelial cells compared to healthy subjects, with this reduction correlating with the clinical severity of the disease [66]. Furthermore, the authors also highlighted a significant decrease in subbasal nerve density, as well as an increase in dendritic cell density, suggesting the concurrent involvement of inflammatory and neurotrophic mechanisms [66].

Similarly, Kheirkhah et al. demonstrated in a longitudinal study that patients with dry eye experience an accelerated loss of corneal endothelial cells over time, exceeding the rate associated with physiological aging. This loss was more pronounced in patients with reduced baseline subbasal nerve density, suggesting the involvement of a neurotrophic mechanism in endothelial damage [69].

These findings can be explained by the pathophysiological mechanisms involved in dry eye syndrome. Alterations in tight junction proteins, such as zonula occludens-1 (ZO-1) and occludin, under inflammatory conditions can compromise the integrity of the corneal barrier. Furthermore, impairment of the neurocorneal unit, including the corneal nerves and their associated neuropeptides, can reduce trophic support and disrupt tissue homeostasis [65].

In addition, hyperosmolarity and epithelial stress activate inflammatory pathways and lead to the release of cytokines that sustain ocular surface inflammation. At the same time, reduced corneal innervation and altered neuroimmune interactions can affect tissue homeostasis. In this context, damage to the corneal endothelium could be the combined result of chronic inflammation and reduced neurotrophic support [65,66,69,70].

Together, these findings suggest that autoimmune and immune-mediated disorders may influence corneal endothelial integrity through multiple mechanisms, including chronic inflammation, immune-mediated cytotoxicity, neurotrophic impairment and ocular surface instability.

### 3.5. Hypoxic and Pulmonary Disorders

Under hypoxic conditions, corneal endothelial function may be impaired by altering the ion transport mechanisms involved in maintaining corneal transparency. Reduced ATP production decreases the activity of the Na^+^/K^+^-ATPase pump and bicarbonate transport, which diminishes the pumping capacity of the endothelium. As a result, fluid can accumulate in the stroma and corneal edema can occur. At the same time, morphological changes in endothelial cells may occur, including polymegathism and a decrease in the proportion of hexagonal cells [71,72,73].

In a study by Soler et al., patients with chronic obstructive pulmonary disease had lower endothelial density and cellular morphological changes compared to individuals without this condition. The study results also showed that, after cataract surgery, patients more frequently presented with transient corneal edema, suggesting an increased susceptibility of the corneal endothelium to intraocular surgical stress [74]. Similar results were reported by Songur et al., who observed lower endothelial density, a reduced percentage of hexagonal cells, and a higher coefficient of variation in chronic obstructive pulmonary disease (COPD) patients compared to the control group [75]. Comparable results were also described by Tașlı et al., who highlighted a decrease in cell density and the proportion of hexagonal cells, associated with an increase in the coefficient of variation, changes that become more pronounced with the severity of the disease [76].

Another clinical model characterized by repeated episodes of systemic hypoxia is obstructive sleep apnea syndrome (OSA). In their study, Bojarun et al. evaluated corneal parameters in patients with OSA compared to healthy subjects. The results showed significant differences in endothelial cell density as well as changes in central corneal thickness. The authors also observed correlations between endothelial density, corneal thickness, and disease severity, as well as with the minimum peripheral oxygen saturation value. These results suggest that repeated episodes of hypoxemia may influence the structure and function of the corneal endothelium [77].

Chronic exposure to hypoxia can also occur in high-altitude environments. In a recent study, Zhu and colleagues compared corneal endothelial parameters in patients living in high-altitude regions and found that endothelial cell density and coefficient of variation did not differ significantly from those of patients in lowland areas. However, the proportion of hexagonal cells was higher in patients from high-altitude regions, suggesting the possibility of compensatory mechanisms of the corneal endothelium under conditions of chronic hypoxia [78].

These findings suggest that systemic hypoxia affects the corneal endothelium primarily by reducing ATP production and disrupting ion transport. At the same time, repeated episodes of hypoxemia can induce oxidative stress and disrupt cellular metabolic balance. These mechanisms contribute to the decline in pump function and the morphological changes observed in the corneal endothelium.

### 3.6. Extracellular Matrix-Related Systemic Disorders

- Pseudoexfoliation syndrome (PEX)

PEX is a systemic condition associated with aging, characterized by the accumulation of abnormal extracellular fibrillar material in various tissues, with predominant involvement of the ocular structures, especially those in the anterior segment [79]. The changes in the corneal endothelium observed in PEX are related to alterations in the intraocular environment. In this condition, the blood–aqueous barrier loses its integrity, and the composition of the aqueous humor changes, with an increase in flare in the anterior chamber. These changes can disrupt the metabolic balance of the corneal endothelium and induce hypoxia and cellular stress, which over time promotes deterioration of the structure and function of endothelial cells [80]. Furthermore, pseudoexfoliative material can deposit on the surface of the corneal endothelium and become incorporated into the posterior portion of Descemet’s membrane. These deposits can affect the structural integrity of the endothelium, reduce pump function, and contribute to the early onset of corneal decompensation [81].

Numerous studies have confirmed that PEX is associated with significant changes in the corneal endothelium. A reduction in endothelial cell density has been observed in patients with PEX compared to healthy subjects, indicating that the endothelium is frequently affected [82,83,84,85,86,87,88]. In addition, morphological changes have been reported, such as increased cell size variability and decreased proportion of hexagonal cells, reflecting the compensatory response of the endothelium to cell loss [89,90,91]. These alterations appear to be more pronounced in patients with pseudoexfoliative glaucoma, suggesting that disease progression and elevated intraocular pressure may exacerbate endothelial damage [92].

Together, these data suggest that pseudoexfoliation syndrome may significantly affect corneal endothelial integrity, both through mechanisms related to the disease itself and through the effects of increased intraocular pressure, contributing to progressive deterioration of endothelial function. These changes may also increase the susceptibility of the corneal endothelium to intraocular surgical stress, particularly in the context of cataract surgery.

## 4. Age-Related Changes

Corneal aging is a complex, multifactorial process characterized by progressive structural and functional changes in all layers of the cornea, including the endothelium [93]. With age, the density of corneal endothelial cells progressively decreases, accompanied by compensatory morphological changes, such as increased variability in cell size and a reduced proportion of hexagonal cells [94]. These structural adaptations reflect cellular enlargement and may disrupt intercellular organization and communication, ultimately impairing the maintenance of corneal hydration and optical properties [95,96]. In addition, aging is also associated with a progressive decline in the metabolic and functional activity of endothelial cells. Recent studies have highlighted the presence of regional heterogeneity, with the peripheral endothelium exhibiting higher metabolic activity compared to the central endothelium, an activity that tends to decrease with age [97]. This progressive attenuation of regional metabolic differences suggests a gradual loss of functional specialization within the corneal endothelium. At the molecular level, aging is also associated with downregulation of pathways involved in metabolism, ion homeostasis, and cell-cycle regulation, further supporting the concept of progressive endothelial functional deterioration [98].

Together, these structural, morphological, and molecular changes highlight the complex impact of aging on corneal endothelial integrity and function.

## 5. Conclusions and Future Directions

**Figure 2:**
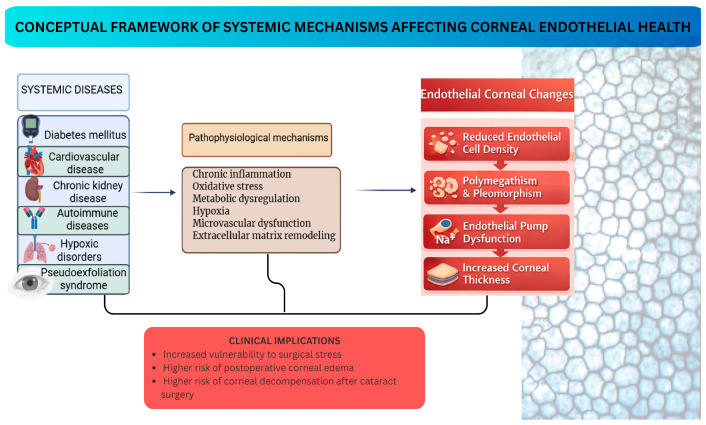
Systemic diseases activate common biological pathways that damage corneal endothelial cells and reduce endothelial reserve, increasing the risk of postoperative complications.

The integrity of the corneal endothelium depends on both local ocular factors and the patient’s overall health. Analyzed data of this review show that numerous systemic diseases are associated with structural and functional changes in corneal endothelial cells. Diabetes mellitus, cardiovascular disease, chronic kidney disease, autoimmune diseases, hypoxic conditions, and pseudoexfoliation show significant associations with signs of endothelial stress. In most of these conditions, the corneal endothelium shows similar changes: decreased cell density, increased cell size variability, decreased percentage of hexagonal cells, and increased central corneal thickness. These changes reflect a reduction in endothelial reserve and a decrease in the cornea’s ability to tolerate additional stress (Figure 2).

With regard to pathophysiological mechanisms, studies show the existence of common pathways of endothelial damage. Chronic inflammation, oxidative stress, metabolic disorders, hypoxia, and microvascular dysfunction contribute to endothelial cell damage, affecting the activity of the Na^+^/K^+^-ATPase pump, altering cellular metabolism, and consequently accelerating progressive cell loss. These observations have direct clinical implications: patients with systemic diseases may present subclinical endothelial vulnerability even in the absence of obvious corneal pathology.

Preoperative evaluation must go beyond the standard eye exam. A detailed systemic history, assessment of metabolic and vascular comorbidities, and analysis of the corneal endothelium by specular microscopy are essential in patients with systemic pathologies. Identifying reduced endothelial reserve prior to surgery allows for safer surgical planning and a more realistic assessment of postoperative risks. These data support the need for an integrated approach, in which corneal endothelium assessment is performed in the context of the patient’s overall health.

## Data Availability

No new data were created or analyzed in this study.

## Abbreviations

The following abbreviations are used in this manuscript:

CECs	corneal endothelial cells (CECs)
µm	micrometers
CCT	central corneal thickness
HbA1c	glycated hemoglobin
CKD	chronic kidney disease
GO	Graves’ orbitopathy
CV	coefficient of variation
OSA	obstructive sleep apnea syndrome
PEX	pseudoexfoliation syndrome
ATP	adenosine triphosphate
COPD	chronic obstructive pulmonary disease
ROS	reactive oxygen species
